# Prevalence and correlates of dyslipidemia in first-episode and drug-naïve major depressive disorder patients with comorbid abnormal glucose metabolism: Sex differences

**DOI:** 10.3389/fpsyt.2023.1101865

**Published:** 2023-01-30

**Authors:** Quanfeng Zhu, Yali Zheng, XiaoE Lang, Zhengchuang Fu, Peng Zhang, Guojun Jiang, Xiangyang Zhang

**Affiliations:** ^1^Graduate School of Zhejiang Chinese Medical University, Hangzhou, China; ^2^Affiliated Xiaoshan Hospital, Hangzhou Normal University, Hangzhou, China; ^3^Department of Psychiatry, First Hospital/First Clinical Medical College of Shanxi Medical University, Taiyuan, China; ^4^Chinese Academy of Sciences (CAS), Key Laboratory of Mental Health, Institute of Psychology, Beijing, China; ^5^Department of Psychology, University of Chinese Academy of Sciences, Beijing, China

**Keywords:** major depressive disorder, dysglycemia, dyslipidemia, first-episode, drug-naïve

## Abstract

**Background:**

Lipid metabolism is associated with glucose metabolism, but whether there are variations between sexes in risk factors and prevalence of abnormal lipid metabolism in major depressive disorder (MDD) patients with glucose metabolism abnormalities remains ambiguous. In the present study, the frequency and risk factors of dyslipidemia in first-episode and drug-naïve (FEDN) MDD patients with dysglycemia were examined according to sex.

**Methods:**

One thousand seven hundred and eighteen FEDN MDD patients were recruited and their demographic data, clinical data, various biochemical indicators and scale assessment scores including 17-item Hamilton Rating Scale for Depression (HAMD-17), 14-item Hamilton Anxiety Rating Scale (HAMA-14), and positive subscale of the Positive and Negative Syndrome Scale (PANSS) were collected.

**Results:**

The prevalence of abnormal lipid metabolism in both male and female MDD patients with abnormal glucose metabolism was higher than that in patients without abnormal glucose metabolism. Among male MDD patients with abnormal glucose metabolism, TC was positively correlated with HAMD score, TSH and TgAb levels, but negatively correlated with PANSS positive subscale scores. LDL-C was positively correlated with TSH and BMI, but negatively correlated with PANSS positive subscale scores. HDL-C was negatively correlated with TSH levels. Among females, TC was positively correlated with HAMD score, TSH, and BMI, but negatively correlated with PANSS positive subscale score. LDL-C was positively correlated with HADM score and negatively correlated with FT3 level. HDL-C was negatively correlated with TSH and BMI levels.

**Conclusion:**

There are sex differences in the correlated factors of lipid markers in MDD patients with impaired glucose.

## 1. Introduction

Major depressive disorder (MDD), one of the most prevalent mental diseases, is a significant global source of disability and places a significant economic burden on society as a whole (1). MDD can depress patients’ mood and severely impair their cognitive function, social skills, and sleep (2). The exact etiology of MDD is unknown, and current research still cannot accurately and independently explain its pathogenesis. It is generally accepted that MDD is caused by social, psychological, and physiological causes, with genetics accounting for approximately 35% of the cases (2).

Epidemiological studies in several countries have shown a high prevalence of MDD. In a large national comorbidity survey in the United States, the lifetime prevalence of MDD reached 20.6% (3). At the same time, treatment rates are generally low in patients with MDD. A large epidemiological survey in China showed that less than 10% of MDD patients are adequately treated (4). On the other hand, about 20–40% of treated patients do not respond significantly to antidepressants (5). In conclusion, MDD has become a serious social problem due to its high morbidity, disability, treatment difficulties, high relapse rate, and relatively low treatment rate.

Patients with MDD have a high prevalence of abnormal glucose and lipid metabolism, which may be related to their antidepressant medication and usual poor diet (6). On the other hand, depression and abnormal glucose and lipid metabolism may share a common emergency system response, such as the hypothalamic-pituitary-adrenal axis, which also predispose MDD patients to lipid metabolism disorders (7). In addition, some studies have also found that suicidal tendencies in MDD patients may be related to their blood glucose levels, which are higher in suicidal MDD patients (8, 9). The findings of Watson et al. suggest a strong association between insulin resistance and depression by exploring the relationship between three surrogate measures of insulin resistance and the incidence of MDD. They found that the higher the three indexes, the higher the prevalence of MDD, and all three surrogate measures can positively predict the occurrence of major depression (10). Steiner et al. also revealed a possible causal relationship between first-episode depression and long-term impaired glucose/insulin homeostasis (11). Some studies suggest that metabolic risk factors such as hyperlipidemia can increase the risk of depression, and lipid metabolism disorders may accelerate the development of depression-like behaviors (12, 13).

Many studies have revealed sex differences in depression. For example, some studies have confirmed that depression is more common in women than in males, but men have higher rates of suicide than women (14, 15). Li et al. showed that women with depression have higher BMI and total adiposity than men, while men have higher visceral adiposity (16). In addition, there are significant sex differences in the incidence, symptom presentation, and treatment response of depression, and Labonté’s team found that these sex differences in depression may be associated with sex differences at the transcriptional level (17).

Sexes differ significantly in impaired glucose metabolism. Impaired fasting glucose is more common in males with prediabetes syndrome, but impaired glucose tolerance is more common in women. Globally, males are more likely than females to have type 2 diabetes. Also, men are more likely to develop diabetes before puberty, while women are more likely to develop diabetes after menopause (18). And there are also sex differences in lipid metabolism. For example, Magkos and Mittendorfer found that women had lower triglyceride concentrations than men (19). There are sex differences in both cholesterol and triglyceride metabolism, which may be related to the action of endogenous sex hormones (20).

However, to our knowledge, there are no reports on whether the prevalence and risk factors for abnormal lipid metabolism differ between males and females in MDD patients with dysglycemia. The aim of the present study was to explore sex differences in the prevalence of dyslipidemia and correlated factors of lipid markers in MDD patients with dysglycemia.

## 2. Materials and methods

### 2.1. Subjects

Cross-sectional research was used in this study. One thousand seven hundred and eighteen patients were recruited by the psychiatric department of the First Hospital of Shanxi Medical University from 2015 to 2017. The recruitment criteria were as follows: (1) age range: 18–60; (2) conforming to the diagnostic criteria for MDD in the Diagnostic and Statistical Manual of Mental Disorders, 4th edition (DSM-IV); (3) first-episode and drug-naïve patients who had not received any antipsychotic or antidepressant medication; and (4) Chinese Han nationality. Exclusion criteria were as follows: (1) diagnosed with a mental illness other than MDD; (2) any organic brain disease; (3) history of drug dependence and alcohol dependence; (4) persistent infection, ongoing immunosuppressive therapy, or other serious physical illness; and (5) pregnant or breastfeeding women.

This study was approved by the Institutional Review Board of the First Hospital of Shanxi Medical University (No. 2016-Y27). A written informed consent form was signed by each patient after they had been apprised of the study’s procedures.

### 2.2. Demographic data

In this study, basic information about each patient was collected by a trained physician in the form of a questionnaire, including age, duration of illness, sex, marriage status, and educational background. Body mass index (BMI) was calculated for each patient by dividing weight (kg) by the square of height (m^2^). To ensure the accuracy of the information, the information was checked with each patient’s guardians or relatives.

### 2.3. Clinical assessment

To gauge the severity of patients’ depression, the 17-item Hamilton Depression Scale (HAMD-17) was employed, which has been demonstrated to have strong reliability and validity among Chinese population (21). In current research, a HAMD-17 score ≥ 24 was used as an evaluation criterion for MDD (22, 23).

In order to gauge the patients’ level of anxiety, the 14-item Hamilton Anxiety Rating Scale (HAMA-14) was employed, which has been proved to have good reliability and validity among Chinese population, too (24). The anxiety level of patients was classified according to the total HAMA score as follows: (1) no anxiety: HAMA score ≤ 7; (2) mild anxiety: 8 ≤ HAMA score ≤ 14; (3) moderate anxiety: 15 ≤ HAMA score ≤ 23; and (4) severe anxiety: HAMA score ≥ 24 (25).

To evaluate psychotic symptoms, we adopted the positive subscale of the Positive and Negative Syndrome Scale (PANSS) (8, 26). Patients were deemed to exhibit psychotic symptoms if they had a positive subscale score of 15 or above (27, 28).

In addition, patients were also investigated for suicidal behavior in the form of a questionnaire. All of the above patient assessment procedures were done separately by two trained physicians, which ensured the reliability of the scoring results.

### 2.4. Measurement of physical and biochemical parameters

All patients were asked to fast after 8 p.m. the previous night and to complete venous blood collection and blood pressure measurement between 6 and 8 a.m. the following morning. All collected blood samples were taken immediately to the hospital laboratory center and tested by 11 a.m. The biochemical parameters were tested included fasting blood glucose (FBG), total serum cholesterol (TC), triglycerides (TG), and high-density lipoprotein cholesterol (HDL-C). low-density lipoprotein cholesterol (LDL-C), thyroid stimulating hormone (TSH), free triiodothyronine (FT3), free thyroxine (FT4), anti-thyroglobulin antibody (TGAb), and thyroid peroxidase antibody (TPOAb). FBG, TC, TG, HDL-C, and LDL-C were measured by ARCHITECT C8000 System (Abbott Laboratories, Irving, TX, USA). Blood pressure was measured by Omron HBP-1300 electronic manometer. FT3, FT4, TSH, TgAb, and TPOAb were detected by Roche C6000 Electrochemiluminescence Immunoassay Analyzer (Roche Diagnostics, Indianapolis, IN, USA). The diagnostic criteria were as follows: (1) abnormal glucose metabolism: FBG > 6.1 mmol/l; (2) abnormal lipid metabolism: TC ≥ 5.2 mmol/l or TG ≥ 1.7 mmol/l or LDL-C ≥ 3.4 mmol/l or HDL-C < 1.0 mmol/l (29); (3) hypertension: diastolic blood pressure ≥ 90 mmHg or systolic blood pressure ≥ 140 mmHg.

### 2.5. Statistical analysis

All statistical analysis for this study was run on SPSS 25.0. The Shapiro–Wilk Test was applied to assess the normality of all continuous variables. All hierarchical variables and non-normally distributed continuous variables were subjected to the Mann–Whitney *U* Test, while normally distributed continuous variables were subjected to the Analysis of Variance (ANOVA). All categorical variables were subjected to the Chi-Square Test. Univariate analysis was performed with the presence of abnormal glucose metabolism as the dependent variable in male and female patients to explore the sex differences in the prevalence and risk factors of depression patients with abnormal glucose metabolism. Finally, we performed multiple linear regression with each of the four lipid indicators (TC, TG, LDL-C, HDL-C) as dependent variables to analyze their correlation with clinical and biochemical indicators. The threshold for statistical significance of the results was *P* < 0.05.

## 3. Results

### 3.1. Sex differences in prevalence of abnormal lipid metabolism in MDD patients with and without abnormal glucose metabolism

Between male and female MDD patients, there were no appreciable differences in the prevalence of impaired glucose metabolism, which was 12.59% (74/588) and 14.16% (160/1130), respectively. According to Table 1, male and female MDD patients with glucose metabolism abnormalities had higher HAMD score, HAMA score, PANSS positive subscale score, TSH, TC, TG, LDL-C, systolic and diastolic blood pressure levels, as well as greater rates of psychotic symptoms, abnormal lipid metabolism, and hypertension but lower HDL-C levels compared with patients of the same sex without glucose metabolism abnormalities.

**TABLE 1:** Sociodemographic and clinical characteristics of male and female MDD patients with and without abnormal glucose metabolism.

Variable	Male				Female			
	MDD comorbidities abnormal glucose metabolism (*N* = 74)	MDD without abnormal glucose metabolism (*N* = 514)	F/χ^2^/Z	*P*	MDD comorbidities abnormal glucose metabolism (*N* = 160)	MDD without abnormal glucose metabolism (*N* = 970)	F/χ^2^/Z	*P*
Age	36.5 (23∼46.25)	30 (21∼42.25)	-2.00	0.046	35.5 (24∼48)	35 (24.75∼47)	-0.09	0.927
Onset age	36 (23∼46.25)	30 (21∼42)	-2.10	0.036	35 (24∼47)	35 (24∼46)	-0.07	0.946
Education			-0.51	0.613			-1.92	0.055
Junior high school, n (%)	21 (28.4%)	104 (20.2%)			48 (30.0%)	240 (24.7%)		
High school, n (%)	28 (37.8%)	243 (47.3%)			72 (45.0%)	417 (43.0%)		
University degree, n (%)	20 (27.0%)	150 (29.2%)			31 (19.4%)	248 (25.6%)		
Master’s degree, n (%)	5 (6.8%)	17 (3.3%)			9 (5.6%)	65 (6.7%)		
Marital status			0.93	0.333			0.07	0.799
Single, n (%)	21 (28.4%)	175 (34.0%)			42 (26.3%)	264 (27.2%)		
Married, n (%)	53 (71.6%)	339 (66.0%)			118 (73.7%)	706 (72.8%)		
Suicide attempters			16.70	<0.001			23.19	<0.001
Yes, n (%)	27 (36.5%)	85 (16.5%)			56 (35.0%)	178 (18.4%)		
No, n (%)	47 (63.5%)	429 (83.5%)			104 (65.0%)	792 (81.6%)		
Anxiety levels			-1.39	0.164			-5.09	<0.001
No anxiety, n (%)	0	0			0	0		
Mild anxiety, n (%)	0	1 (0.2%)			0	5 (0.5%)		
Moderate anxiety, n (%)	57 (77.0%)	428 (83.3%)			106 (66.3%)	802 (82.7%)		
Severe anxiety, n (%)	17 (23.0%)	85 (16.5%)			54 (33.7%)	163 (16.8%)		
Psychotic symptoms			10.55	0.001			13.47	<0.001
Yes, n (%)	13 (17.6%)	34 (6.6%)			31 (19.4%)	93 (9.6%)		
No, n (%)	61 (82.4%)	480 (93.4%)			129 (80.6%)	877 (90.4%)		
Abnormal lipid metabolism			6.25	0.012			15.93	<0.001
Yes, n (%)	67 (90.5%)	401 (78.0%)			149 (93.1%)	776 (80.0%)		
No, n (%)	7 (9.5%)	113 (22.0%)			11 (6.9%)	194 (20.0%)		
Hypertension			4.89	0.027			11.40	<0.001
Yes, n (%)	8 (10.8%)	21 (4.1%)			18 (11.3%)	45 (4.6%)		
No, n (%)	66 (89.2%)	493 (95.9%)			142 (88.7%)	925 (95.4%)		
HAMD	31 (28.75∼33)	30 (28∼32)	-2.28	0.023	32 (30∼33)	30 (28∼32)	-6.09	<0.001
HAMA	21 (19∼23)	20 (18∼23)	-2.41	0.016	22 (20∼24)	21 (18∼23)	-4.87	<0.001
PANSS positive subscale score	7 (7∼10)	7 (7∼7)	-2.97	0.003	7 (7∼11)	7 (7∼7)	-4.95	<0.001
BMI, kg/m^2^	24.58 (23.32∼26.01)	24.19 (23.15∼25.60)	-1.06	0.29	24.37 (23.39∼25.98)	24.22 (23.24∼25.50)	-1.03	0.304
Systolic BP, mmHg	122 (117.75∼128)	118 (110.75∼125)	-3.40	<0.001	124.5 (118∼130)	120 (111∼126)	-5.37	<0.001
Diastolic BP, mmHg	78 (72∼84)	75 (70∼80)	-2.58	<0.001	78 (74∼83)	75 (70∼80)	-5.61	<0.001
TC, mmol/L	5.57 (4.82∼6.35)	5.11 (4.44∼5.91)	-3.64	<0.001	5.83 (5.12∼6.78)	5.15 (4.39∼5.84)	-6.85	<0.001
TG, mmol/L	2.48 (1.46∼3.21)	1.89 (1.40∼2.68)	-2.68	0.007	2.28 (1.65∼2.96)	1.97 (1.36∼2.75)	-3.69	<0.001
HDL-C, mmol/L	1.21 (0.87∼1.34)	1.25 (1.05∼1.43)	-1.99	0.047	1.1 (0.86∼1.27)	1.24 (1.04∼1.43)	-5.10	<0.001
LDL-C, mmol/L	3.21 ± 0.90	2.93 ± 0.84	0.06	0.007	3.3 (2.7∼3.89)	2.9 (2.3∼3.4425)	-4.83	<0.001
FBG, mmol/L	6.36 (6.20∼6.57)	5.19 (4.86∼5.60)	-13.92	<0.001	6.45 (6.24∼6.63)	5.21 (4.89∼5.6)	-20.29	<0.001
TSH, uIU/mL	6.87 (5.15∼8.87)	4.72 (2.90∼6.36)	-6.16	<0.001	7.47 (5.85∼9.37)	4.62 (2.97∼6.24)	-10.90	<0.001
FT3, pmol/L	5.02 (4.42∼5.33)	5.01 (4.42∼5.48)	-0.29	0.773	4.86 (4.26∼5.31)	4.87 (4.39∼5.4)	-1.47	0.143
FT4, pmol/L	16.55 (14.43∼18.87)	16.44 (14.41∼18.84)	-0.06	0.954	16.78 (14.63∼18.89)	16.53 (14.33∼18.62)	-1.34	0.180
TgAb, IU/L	20.23 (15.10∼81.94)	21.19 (14.13∼35.25)	-0.59	0.557	24.18 (15.72∼115.74)	21.29 (14.32∼39.92)	-3.11	0.002
TPOAb, IU/L	16.97 (12.53∼88.86)	16.81 (12.43∼32.35)	-0.85	0.397	22.77 (12.70∼97.73)	17.40 (12.21∼33.38)	-3.24	0.001

### 3.2. Prevalence of abnormal lipid metabolism in MDD patients with and without abnormal glucose metabolism

The probability of abnormal lipid metabolism in male MDD patients with and without comorbid abnormal glucose metabolism was 90.54% (67/74) and 78.02% (401/514), respectively, with significant differences (*P* < 0.05). Similarly, the probability of abnormal lipid metabolism in female MDD patients with and without abnormal glucose metabolism was 93.13% (149/160) and 80.00% (776/970), respectively, with a significant difference (*P* < 0.001).

### 3.3. Sex differences in factors influencing lipid indicators in MDD patients with abnormal glucose metabolism

According to Supplementary Tables 1–4, in male MDD patients with abnormal glucose metabolism, HAMD score (β = 0.40, *P* < 0.05, 95% CI: 0.06–0.23, VIF = 2.06) and PANSS positive subscale score (β = −0.46, *P* < 0.05, 95% CI: −0.15 to −0.04, VIF = 2.36), TSH (β = 0.45, *P* < 0.001, 95% CI: 0.09–0.29, VIF = 2.13), and TgAb (β = 0.24, *P* < 0.05, 95% CI: 0.0002–0.002, VIF = 1.54) were significantly associated with TC. PANSS positive subscale score (β = −0.33, *P* < 0.05, 95% CI: −0.11 to −0.001, VIF = 2.36), TSH (β = 0.41, *P* < 0.05, 95% CI: 0.03–0.24, VIF = 2.13), and BMI (β = 0.31, *P* < 0.05, 95% CI: 0.02–0.19, VIF = 1.26) were significantly associated with LDL-C. TSH (β = −0.37, *P* < 0.05, 95% CI: −0.08 to −0.008, VIF = 2.13) was significantly associated with HDL-C. There was no significant correlation between TG and any of the indicators.

However, in female MDD patients with abnormal glucose metabolism, HAMD score (β = 0.54, *P* < 0.001, 95% CI: 0.16–0.29, VIF = 1.66), PANSS positive subscale score (β = −0.18, *P* < 0.05, 95%CI: −0.07 to −0.002, VIF = 2.11), TSH (β = 0.31, *P* < 0.001, 95% CI: 0.06–0.20, VIF = 1.82), and BMI (β = 0.16, *P* < 0.01, 95% CI: 0.03–0.17, VIF = 1.06) were significantly correlated with TC. HAMD score (β = 0.39, *P* < 0.001, 95% CI: 0.07–0.20, VIF = 1.66) and FT3 (β = −0.16, *P* < 0.05, 95% CI: −0.45 to −0.007, VIF = 1.15) were significantly correlated with LDL-C. TSH (β = −0.25, *P* < 0.05, 95% CI: −0.05 to −0.005, VIF = 1.82) and BMI (β = −0.18, *P* < 0.05, 95% CI: −0.05 to −0.003, VIF = 1.06) were significantly associated with HDL-C. And BMI (β = 0.178, *P* < 0.05, 95% CI: 0.01–0.18, VIF = 1.06) was significantly associated with TG.

## 4. Discussion

To the best of our knowledge, the present study is the first large-scale study to compare sex differences in the prevalence and associated factors of abnormal lipid metabolism in FEDN MDD patients with abnormal glucose metabolism. We found that in FEDN MDD patients, the prevalence of abnormal lipid metabolism was significantly higher in those with abnormal glucose metabolism, and there was no sex difference.

Since different disease progression and antipsychotics or antidepressants taken by depressed patients have significant effects on blood glucose and blood lipids, patients with FEDN MDD were included in the present study to exclude drug interference on blood glucose and blood lipids.

Major depressive disorder patients with abnormal glucose metabolism have a higher prevalence of abnormal lipid metabolism, which is consistent with most previous findings. Many studies have confirmed that dyslipidemia is very common in patients with type 2 diabetes, mainly manifested as high TG and low HDL-C, and there is little sex difference. Insulin resistance may be one of the main causes of abnormal lipid metabolism in patients with abnormal glucose metabolism (30, 31).

The association between BMI and abnormal lipid metabolism is easy to understand. The higher the BMI, the more severe the obesity. There is a close relationship between obesity and abnormal lipid metabolism. Many studies have revealed a close correlation between high BMI and abnormal lipid metabolism (32, 33). In the present study, we found a significant association between high BMI and abnormal lipid metabolism only in female MDD patients with abnormal glucose metabolism. The study of Zheng found that in the Chinese children population, the correlation between BMI value and dyslipidemia in males was more significant than that in females (34). The reason for these sex differences may be related to sample heterogeneity.

Many studies have pointed out that there is a close relationship between thyroid function and lipid metabolism, and dyslipidemia may be aggravated with the increase of TSH level. At the same time, patients with autoimmune thyroid disease are more likely to have abnormal lipid metabolism (35–38). It has been suggested that triiodothyronine may affect lipid turnover in adipocytes through the hypothalamus and other central nervous systems, and that TSH can regulate lipid metabolism by controlling lipogenesis and breakdown (36).

Some previous studies have also reported sex differences in the association between thyroid function and abnormal lipid metabolism. For example, the study by Wu et al. revealed that metabolic disorders were associated with increased positive levels of thyroid autoantibodies with sex differences. Females with TgAb^+^ have higher TC and LDL-C levels (39). While Zhang’s group found that TgAb^+^ may be a protective factor against hypertriglyceridemia in females, but not in males (40). In the present study, the association between dyslipidemia and thyroid function in MDD patients with abnormal glucose metabolism was different by sex.

We suggest that this sex difference may be due to the following reasons. On the one hand, it may be because of the sex difference in leptin levels in the body. Leptin plays an important role in glucose and lipid metabolism. Leptin can regulate glucose metabolism through the mediation of central and peripheral tissues and under the influence of the activation of insulin signaling pathway. In addition, leptin in central and peripheral tissues can also reduce the accumulation of ectopic fat and play a role in regulating lipid metabolism (41). Several studies have revealed large sex differences in the magnitude of leptin’s effects (41). The second reason may be due to sex differences in endogenous sex hormones. Many studies have confirmed that endogenous sex hormones have profound effects on lipid metabolism. TC and TG are all regulated by endogenous androgens and estrogens, and this complex regulatory mechanism is carried out in a variety of tissues and mediated effects (20, 42). And the third reason may be due to the sex differences in liver gene expression. Several studies have found that there are obvious sex differences in the expression of more than 1,000 human liver genes, among which the sex differences in the expression of genes related to lipid metabolism may also be one of the important reasons for the sex differences in dyslipidemia related factors in MDD patients with abnormal glucose metabolism (42).

Our study also has some limitations. First, all patients recruited in this study were from the same neighboring area, so the results may not be representative of other areas. Second, the present research was cross-sectional in nature and could not explain the causal relationship between abnormal lipid metabolism and various factors. Third, adverse lifestyle factors such as lack of exercise and adverse dietary habits such as binge eating in MDD patients can have a serious impact on glucose and lipid metabolism. Because these factors are difficult to quantify, the effects of diet and lifestyle on glucose and lipid metabolism and other clinical indicators in MDD patients were not investigated in this study. Fourth, in our present study, among MDD patients with abnormal glucose metabolism, there was a large difference in the number of patients with and without abnormal lipid metabolism, which may affect the statistical results. Finally, this study did not establish a healthy control group to explore the differences in glucolipid metabolism between male and female MDD patients and healthy controls.

## Data availability statement

The raw data supporting the conclusions of this article will be made available by the authors, without undue reservation.

## Ethics statement

The studies involving human participants were reviewed and approved by Institutional Review Board of the First Hospital of Shanxi Medical University. The patients/participants provided their written informed consent to participate in this study.

## Author contributions

QZ: data processing and thesis writing. YZ: thesis writing and modification. XL: data collection. ZF and PZ: data processing. GJ: thesis guidance. XZ: project design. All authors contributed to the article and approved the submitted version.

## References

[1] Rottenberg J. Emotions in depression: what do we really know? *Annu Rev Clin Psychol.* (2017) 13:241–63. 10.1146/annurev-clinpsy-032816-045252 28375721

[2] Otte C Gold S Penninx B Pariante C Etkin A Fava M Major depressive disorder. *Nat Rev Dis Primers.* (2016) 2:16065. 10.1038/nrdp.2016.65 27629598

[3] Hasin D Sarvet A Meyers J Saha T Ruan W Stohl M Epidemiology of adult DSM-5 major depressive disorder and its specifiers in the united states. *JAMA Psychiatry.* (2018) 75:336–46. 10.1001/jamapsychiatry.2017.4602 29450462 PMC5875313

[4] Lu J Xu X Huang Y Li T Ma C Xu G Prevalence of depressive disorders and treatment in China: a cross-sectional epidemiological study. *Lancet Psychiatry.* (2021) 8:981–90. 10.1016/S2215-0366(21)00251-0 34559991

[5] Touloumis C. The burden and the challenge of treatment-resistant depression. *Psychiatriki.* (2021) 32:11–4. 10.22365/jpsych.2021.046 34990376

[6] Vancampfort D Correll C Wampers M Sienaert P Mitchell A De Herdt A Metabolic syndrome and metabolic abnormalities in patients with major depressive disorder: a meta-analysis of prevalences and moderating variables. *Psychol Med.* (2014) 44:2017–28. 10.1017/S0033291713002778 24262678

[7] Marazziti D Rutigliano G Baroni S Landi P Dell’Osso L. Metabolic syndrome and major depression. *CNS Spectr.* (2014) 19:293–304. 10.1017/S1092852913000667 24103843

[8] Liu W Wu Z Sun M Zhang S Yuan J Zhu D Association between fasting blood glucose and thyroid stimulating hormones and suicidal tendency and disease severity in patients with major depressive disorder. *Bosn J Basic Med Sci.* (2022) 22:635–42. 10.17305/bjbms.2021.6754 35238287 PMC9392982

[9] Koponen H Kautiainen H Leppänen E Mäntyselkä P Vanhala M. Association between suicidal behaviour and impaired glucose metabolism in depressive disorders. *BMC Psychiatry.* (2015) 15:163. 10.1186/s12888-015-0567-x 26199013 PMC4509469

[10] Watson K Simard J Henderson V Nutkiewicz L Lamers F Nasca C Incident major depressive disorder predicted by three measures of insulin resistance: a Dutch cohort study. *Am J Psychiatry.* (2021) 178:914–20. 10.1176/appi.ajp.2021.20101479 34551583

[11] Steiner J Fernandes B Guest P Dobrowolny H Meyer-Lotz G Westphal S Glucose homeostasis in major depression and schizophrenia: a comparison among drug-naïve first-episode patients. *Eur Arch Psychiatry Clin Neurosci.* (2019) 269:373–7. 10.1007/s00406-018-0865-7 29352386

[12] Cho Y Mishiro I Akaki T Akimoto T Fujikawa K. Diseases prevalent before major depressive disorder diagnosis: an exploratory nested case-control study using health insurance-based claims data. *BMJ Open.* (2022) 12:e048233. 10.1136/bmjopen-2020-048233 35168961 PMC8852671

[13] Yu H Qin X Yu Z Chen Y Tang L Shan W. Effects of high-fat diet on the formation of depressive-like behavior in mice. *Food Funct.* (2021) 12:6416–31. 10.1039/d1fo00044f 34076000

[14] Bjerkeset O Romundstad P Gunnell D. Gender differences in the association of mixed anxiety and depression with suicide. *Br J Psychiatry.* (2008) 192:474–5. 10.1192/bjp.bp.107.045203 18515904

[15] Labaka A Goñi-Balentziaga O Lebeña A Pérez-Tejada J. Biological sex differences in depression: a systematic review. *Biol Res Nurs.* (2018) 20:383–92. 10.1177/1099800418776082 29759000

[16] Li L Gower B Shelton R Wu X. Gender-specific relationship between obesity and major depression. *Front Endocrinol.* (2017) 8:292. 10.3389/fendo.2017.00292 29176959 PMC5686049

[17] Labonté B Engmann O Purushothaman I Menard C Wang J Tan C Sex-specific transcriptional signatures in human depression. *Nat Med.* (2017) 23:1102–11. 10.1038/nm.4386 28825715 PMC5734943

[18] Mauvais-Jarvis F. Gender differences in glucose homeostasis and diabetes. *Physiol Behav.* (2018) 187:20–3. 10.1016/j.physbeh.2017.08.016 28843891 PMC5826763

[19] Magkos F Mittendorfer B. Gender differences in lipid metabolism and the effect of obesity. *Obstet Gynecol Clin North Am.* (2009) 36:245–65; vii. 10.1016/j.ogc.2009.03.001 19501312

[20] Palmisano B Zhu L Eckel R Stafford J. Sex differences in lipid and lipoprotein metabolism. *Mol Metab.* (2018) 15:45–55. 10.1016/j.molmet.2018.05.008 29858147 PMC6066747

[21] Sun X Li Y Yu C Li L. [Reliability and validity of depression scales of Chinese version: a systematic review]. *Zhonghua Liu Xing Bing Xue Za Zhi.* (2017) 38:110–6. 10.3760/cma.j.issn.0254-6450.2017.01.021 28100388

[22] Zimmerman M Martinez J Young D Chelminski I Dalrymple K. Severity classification on the Hamilton depression rating scale. *J Affect Disord.* (2013) 150:384–8. 10.1016/j.jad.2013.04.028 23759278

[23] Chen S Li X Lang X Li J Zhang X. Metabolic parameters and thyroid hormones in relation to suicide attempts in patients with first-episode and drug-naive major depressive disorder with comorbid glucose disturbances: a large cross-sectional study. *Eur Arch Psychiatry Clin Neurosci.* (2022). [Epub ahead of print]. 10.1007/s00406-022-01490-w 36127506

[24] Lin G. [Uses of HAMA the rating scale in neurosis]. *Zhonghua Shen Jing Jing Shen Ke Za Zhi.* (1986) 19:342–4. 3582026

[25] Matza L Morlock R Sexton C Malley K Feltner D. Identifying HAM-A cutoffs for mild, moderate, and severe generalized anxiety disorder. *Int J Methods Psychiatr Res.* (2010) 19:223–32. 10.1002/mpr.323 20718076 PMC6878292

[26] Strube W Cimpianu C Ulbrich M Öztürk Ö Schneider-Axmann T Falkai P Unstable belief formation and slowed decision-making: evidence that the jumping-to-conclusions bias in schizophrenia is not linked to impulsive decision-making. *Schizophr Bull.* (2022) 48:347–58. 10.1093/schbul/sbab108 34554260 PMC8886605

[27] Dong R Haque A Wu H Placide J Yu L Zhang X. Sex differences in the association between suicide attempts and glucose disturbances in first-episode and drug naive patients with major depressive disorder. *J Affect Disord.* (2021) 292:559–64. 10.1016/j.jad.2021.05.110 34147968

[28] Kay S Fiszbein A Opler L. The positive and negative syndrome scale (PANSS) for schizophrenia. *Schizophr Bull.* (1987) 13:261–76. 3616518 10.1093/schbul/13.2.261

[29] Joint Committee Issued Chinese Guideline for the Management of Dyslipidemia in Adults. [2016 Chinese guideline for the management of dyslipidemia in adults]. *Zhonghua Xin Xue Guan Bing Za Zhi.* (2016) 44:833–53. 10.3760/cma.j.issn.0253-3758.2016.10.005 27903370

[30] Athyros V Doumas M Imprialos K Stavropoulos K Georgianou E Katsimardou A Diabetes and lipid metabolism. *Hormones (Athens).* (2018) 17:61–7. 10.1007/s42000-018-0014-8 29858856

[31] Taskinen M Borén J. New insights into the pathophysiology of dyslipidemia in type 2 diabetes. *Atherosclerosis.* (2015) 239:483–95. 10.1016/j.atherosclerosis.2015.01.039 25706066

[32] Costa-Urrutia P Colistro V Franco-Trecu V Granados J Álvarez Fariña R Rodríguez-Arellano M. Dyslipidemia, obesity, and ethnicity in Mexican children. *Int J Environ Res Public Health.* (2021) 18:12659. 10.3390/ijerph182312659 34886385 PMC8656470

[33] Yu L Xu X Yu W Chen L Zhang S Li Y The effect of BMI on blood lipids and dyslipidemia in lactating women. *Nutrients.* (2022) 14:5174. 10.3390/nu14235174 36501203 PMC9737353

[34] Zheng W Zhao A Xue Y Zheng Y Chen Y Mu Z Gender and urban-rural difference in anthropometric indices predicting dyslipidemia in Chinese primary school children: a cross-sectional study. *Lipids Health Dis.* (2016) 15:87. 10.1186/s12944-016-0255-y 27129304 PMC4851820

[35] Lei Y Yang J Li H Zhong H Wan Q. Changes in glucose-lipid metabolism, insulin resistance, and inflammatory factors in patients with autoimmune thyroid disease. *J Clin Lab Anal.* (2019) 33:e22929. 10.1002/jcla.22929 31350776 PMC6757119

[36] Walczak K Sieminska L. Obesity and thyroid axis. *Int J Environ Res Public Health.* (2021) 18:9434. 10.3390/ijerph18189434 34574358 PMC8467528

[37] Wang J Zhuang Z Shao C Yu C Wang W Zhang K Assessment of causal association between thyroid function and lipid metabolism: a Mendelian randomization study. *Chin Med J (Engl).* (2021) 134:1064–9. 10.1097/CM9.0000000000001505 33942801 PMC8116035

[38] Delitala A Fanciulli G Maioli M Delitala G. Subclinical hypothyroidism, lipid metabolism and cardiovascular disease. *Eur J Intern Med.* (2017) 38:17–24. 10.1016/j.ejim.2016.12.015 28040402

[39] Wu Y Shi X Tang X Li Y Tong N Wang G The correlation between metabolic disorders and Tpoab/Tgab: a cross-sectional population-based study. *Endocr Pract.* (2020) 26:869–82. 10.4158/EP-2020-0008 33471678

[40] Zhang J Gao Y Li Y Teng D Xue Y Yan L The presence of serum TgAb suggests lower risks for glucose and lipid metabolic disorders in euthyroid general population from a national survey. *Front Endocrinol (Lausanne).* (2020) 11:139. 32256451 10.3389/fendo.2020.00139 PMC7093715

[41] Pereira S Cline D Glavas M Covey S Kieffer T. Tissue-specific effects of leptin on glucose and lipid metabolism. *Endocr Rev.* (2021) 42:1–28. 10.1210/endrev/bnaa027 33150398 PMC7846142

[42] Palmisano B Zhu L Stafford J. Role of estrogens in the regulation of liver lipid metabolism. *Adv Exp Med Biol.* (2017) 1043:227–56. 10.1007/978-3-319-70178-3_12 29224098 PMC5763482

