# Supplementary material for: Prevalence and correlates of dyslipidemia in first-episode and drug-naïve major depressive disorder patients with comorbid abnormal glucose metabolism: Sex differences
Source: Front Psychiatry. 2023 Jan 30;14:1101865. doi: 10.3389/fpsyt.2023.1101865 (PMC9922762; doi:10.3389/fpsyt.2023.1101865)
Supplement: Supplementary file 1 [file Table_1.docx]

Table S1: Related factors of TC in male and female MDD patients with abnormal glucose metabolism

| **Variable** | **Male** | | | | **Female** | | | |
| --- | --- | --- | --- | --- | --- | --- | --- | --- |
|  | **β** | **95% CI** | **P** | **VIF** | **β** | **95% CI** | **P** | **VIF** |
| Age | -0.133 | (-0.032, 0.008) | 0.244 | 1.924 | -0.027 | (-0.016, 0.011) | 0.725 | 1.661 |
| HAMD | 0.401 | (0.061, 0.232) | 0.001 | 2.064 | 0.537 | (0.161, 0.288) | <0.001 | 1.656 |
| HAMA | 0.263 | (-0.006, 0.189) | 0.064 | 2.920 | 0.047 | (-0.040, 0.068) | 0.606 | 2.274 |
| PANSS positive subscale score | -0.456 | (-0.148, -0.043) | <0.001 | 2.358 | -0.182 | (-0.071, -0.002) | 0.038 | 2.108 |
| TSH, uIU/mL | 0.453 | (0.090, 0.290) | <0.001 | 2.126 | 0.306 | (0.061, 0.195) | <0.001 | 1.824 |
| TgAb, IU/L | 0.241 | (0.0002, 0.002) | 0.020 | 1.542 | 0.026 | (-0.0005, 0.001) | 0.701 | 1.292 |
| TPOAb, IU/L | -0.188 | (-0.002, 0.00003) | 0.058 | 1.414 | 0.005 | (-0.001, 0.001) | 0.941 | 1.266 |
| FT3, pmol/L | 0.128 | (-0.126, 0.571) | 0.206 | 1.510 | -0.017 | (-0.249, 0.189) | 0.789 | 1.146 |
| FT4, pmol/L | -0.073 | (-0.098, 0.043) | 0.436 | 1.289 | 0.001 | (-0.047, 0.047) | 0.993 | 1.059 |
| BMI, kg/m^2^ | 0.001 | (-0.080, 0.081) | 0.994 | 1.262 | 0.164 | (0.025, 0.169) | 0.009 | 1.063 |
| Systolic BP, mmHg | 0.004 | (-0.036, 0.037) | 0.981 | 3.405 | -0.126 | (-0.038, 0.010) | 0.239 | 3.156 |
| Diastolic BP, mmHg | 0.034 | (-0.033, 0.044) | 0.779 | 2.186 | 0.161 | (-0.002, 0.057) | 0.065 | 2.088 |
