# Supplementary material for: Prevalence and correlates of dyslipidemia in first-episode and drug-naïve major depressive disorder patients with comorbid abnormal glucose metabolism: Sex differences
Source: Front Psychiatry. 2023 Jan 30;14:1101865. doi: 10.3389/fpsyt.2023.1101865 (PMC9922762; doi:10.3389/fpsyt.2023.1101865)
Supplement: Supplementary file 2 [file Table_2.docx]

Table S2: Related factors of LDL-C in male and female MDD patients with abnormal glucose metabolism

| **Variable** | **Male** | | | | **Female** | | | |
| --- | --- | --- | --- | --- | --- | --- | --- | --- |
|  | **β** | **95% CI** | **P** | **VIF** | **β** | **95% CI** | **P** | **VIF** |
| Age | 0.014 | (-0.02, 0.02) | 0.923 | 1.924 | 0.076 | (-0.008, 0.02) | 0.417 | 1.661 |
| HAMD | 0.190 | (-0.03, 0.15) | 0.219 | 2.064 | 0.394 | (0.07, 0.20) | <0.001 | 1.656 |
| HAMA | -0.039 | (-0.11, 0.09) | 0.831 | 2.920 | 0.121 | (-0.02, 0.08) | 0.270 | 2.274 |
| PANSS positive subscale score | -0.334 | (-0.11, -0.001) | 0.045 | 2.358 | -0.205 | (-0.07, 0.0005) | 0.053 | 2.108 |
| TSH, uIU/mL | 0.408 | (0.03, 0.24) | 0.011 | 2.126 | 0.127 | (-0.02, 0.11) | 0.197 | 1.824 |
| TgAb, IU/L | 0.087 | (-0.0006, 0.001) | 0.513 | 1.542 | 0.011 | (-0.001, 0.001) | 0.895 | 1.292 |
| TPOAb, IU/L | 0.028 | (-0.0009, 0.001) | 0.823 | 1.414 | -0.093 | (-0.001, 0.0002) | 0.258 | 1.266 |
| FT3, pmol/L | -0.249 | (-0.71, 0.02) | 0.061 | 1.510 | -0.158 | (-0.45, -0.007) | 0.043 | 1.146 |
| FT4, pmol/L | 0.181 | (-0.02, 0.13) | 0.139 | 1.289 | 0.013 | (-0.04, 0.05) | 0.862 | 1.059 |
| BMI, kg/m^2^ | 0.308 | (0.02, 0.19) | 0.012 | 1.262 | 0.118 | (-0.01, 0.13) | 0.116 | 1.063 |
| Systolic BP, mmHg | -0.028 | (-0.04, 0.04) | 0.888 | 3.405 | -0.101 | (-0.03, 0.01) | 0.435 | 3.156 |
| Diastolic BP, mmHg | 0.001 | (-0.04, 0.04) | 0.996 | 2.186 | 0.157 | (-0.007, 0.05) | 0.137 | 2.088 |
