# Supplementary material for: Prevalence and correlates of dyslipidemia in first-episode and drug-naïve major depressive disorder patients with comorbid abnormal glucose metabolism: Sex differences
Source: Front Psychiatry. 2023 Jan 30;14:1101865. doi: 10.3389/fpsyt.2023.1101865 (PMC9922762; doi:10.3389/fpsyt.2023.1101865)
Supplement: Supplementary file 3 [file Table_3.docx]

Table S3: Related factors of HDL-C in male and female MDD patients with abnormal glucose metabolism

| **Variable** | **Male** | | | | **Female** | | | |
| --- | --- | --- | --- | --- | --- | --- | --- | --- |
|  | **β** | **95% CI** | **P** | **VIF** | **β** | **95% CI** | **P** | **VIF** |
| Age | 0.020 | (-0.007, 0.008) | 0.890 | 1.924 | 0.150 | (-0.001, 0.008) | 0.125 | 1.661 |
| HAMD | -0.187 | (-0.051, 0.012) | 0.213 | 2.064 | -0.085 | (-0.029, 0.011) | 0.381 | 1.656 |
| HAMA | 0.019 | (-0.034, 0.037) | 0.917 | 2.920 | -0.011 | (-0.018, 0.016) | 0.926 | 2.274 |
| PANSS positive subscale score | 0.124 | (-0.012, 0.027) | 0.437 | 2.358 | 0.021 | (-0.010, 0.012) | 0.845 | 2.108 |
| TSH, uIU/mL | -0.366 | (-0.081, -0.008) | 0.018 | 2.126 | -0.247 | (-0.048, -0.005) | 0.016 | 1.824 |
| TgAb, IU/L | -0.151 | (-0.0005, 0.0001) | 0.244 | 1.542 | -0.073 | (-0.0003, 0.0001) | 0.391 | 1.292 |
| TPOAb, IU/L | 0.140 | (-0.0001, 0.001) | 0.262 | 1.414 | 0.014 | (-0.0002, 0.0002) | 0.871 | 1.266 |
| FT3, pmol/L | -0.070 | (-0.162, 0.092) | 0.586 | 1.510 | -0.079 | (-0.105, 0.036) | 0.329 | 1.146 |
| FT4, pmol/L | -0.195 | (-0.047, 0.004) | 0.103 | 1.289 | 0.018 | (-0.013, 0.017) | 0.815 | 1.059 |
| BMI, kg/m^2^ | 0.161 | (-0.009, 0.050) | 0.172 | 1.262 | -0.175 | (-0.050, -0.003) | 0.025 | 1.063 |
| Systolic BP, mmHg | -0.316 | (-0.025, 0.002) | 0.103 | 3.405 | -0.006 | (-0.008, 0.007) | 0.962 | 3.156 |
| Diastolic BP, mmHg | 0.092 | (-0.010, 0.018) | 0.552 | 2.186 | -0.051 | (-0.012, 0.007) | 0.636 | 2.088 |
