# Supplementary material for: Prevalence and correlates of dyslipidemia in first-episode and drug-naïve major depressive disorder patients with comorbid abnormal glucose metabolism: Sex differences
Source: Front Psychiatry. 2023 Jan 30;14:1101865. doi: 10.3389/fpsyt.2023.1101865 (PMC9922762; doi:10.3389/fpsyt.2023.1101865)
Supplement: Supplementary file 4 [file Table_4.docx]

Table S4: Related factors of TG in male and female MDD patients with abnormal glucose metabolism

| **Variable** | **Male** | | | | **Female** | | | |
| --- | --- | --- | --- | --- | --- | --- | --- | --- |
|  | **β** | **95% CI** | **P** | **VIF** | **β** | **95% CI** | **P** | **VIF** |
| Age | 0.059 | (-0.024, 0.035) | 0.718 | 1.924 | -0.002 | (-0.016, 0.016) | 0.984 | 1.661 |
| HAMD | -0.247 | (-0.216, 0.033) | 0.146 | 2.064 | -0.115 | (-0.116, 0.031) | 0.254 | 1.656 |
| HAMA | 0.027 | (-0.132, 0.151) | 0.895 | 2.920 | 0.010 | (-0.060, 0.065) | 0.934 | 2.274 |
| PANSS positive subscale score | 0.115 | (-0.052, 0.101) | 0.523 | 2.358 | 0.116 | (-0.019, 0.060) | 0.305 | 2.108 |
| TSH, uIU/mL | 0.029 | (-0.022, 0.269) | 0.094 | 2.126 | 0.129 | (-0.029, 0.125) | 0.222 | 1.824 |
| TgAb, IU/L | 0.036 | (-0.001, 0.001) | 0.805 | 1.542 | 0.172 | (-0.00001, 0.001) | 0.054 | 1.292 |
| TPOAb, IU/L | -0.107 | (-0.002, 0.001) | 0.443 | 1.414 | -0.018 | (-0.001, 0.001) | 0.840 | 1.266 |
| FT3, pmol/L | 0.050 | (-0.417, 0.595) | 0.727 | 1.510 | 0.076 | (-0.137, 0.370) | 0.364 | 1.146 |
| FT4, pmol/L | -0.203 | (-0.180, 0.024) | 0.131 | 1.289 | -0.022 | (-0.062, 0.047) | 0.784 | 1.059 |
| BMI, kg/m^2^ | -0.171 | (-0.193, 0.040) | 0.196 | 1.262 | 0.178 | (0.010, 0.177) | 0.028 | 1.063 |
| Systolic BP, mmHg | -0.026 | (-0.057, 0.050) | 0.903 | 3.405 | -0.039 | (-0.031, 0.024) | 0.776 | 3.156 |
| Diastolic BP, mmHg | 0.068 | (-0.044, 0.067) | 0.693 | 2.186 | -0.040 | (-0.040, 0.028) | 0.720 | 2.088 |
